# Supplementary material for: Cytogeography of Naturalized Solidago canadensis Populations in Europe
Source: Plants (Basel). 2023 Mar 1;12(5):1113. doi: 10.3390/plants12051113 (PMC10005290; doi:10.3390/plants12051113)
Supplement: Supplementary file 1 [file plants-12-01113-s001.zip › plants-2196262-supplementary.pdf]

Table S1. Population used for morphological traits

Population No.	Latitude	Longitude	Phenotype identification	Molecular Identification	Ploidy
Introduced range					
CN01	31.13	121.19	*S. canadensis*	*S. canadensis*	6x
CN02	31.15	121.38	*S. canadensis*	*S. canadensis*	4x/6x
CN04	32.03	118.49	*S. canadensis*	*S. canadensis*	4x
CN05	32.11	118.83	*S. canadensis*	*S. canadensis*	4x
CN06	32.09	118.89	*S. canadensis*	*S. canadensis*	6x
CN07	32.30	119.53	*S. canadensis*	*S. canadensis*	6x
CN09	31.49	120.45	*S. canadensis*	*S. canadensis*	6x
CN10	32.02	120.58	*S. canadensis*	*S. canadensis*	6x
CN11	31.93	121.13	*S. canadensis*	*S. canadensis*	4x
CN12	31.53	120.15	*S. canadensis*	*S. canadensis*	6x
CN13	31.50	120.07	*S. canadensis*	*S. canadensis*	4x
CN14	31.02	120.38	*S. canadensis*	*S. canadensis*	6x
CN15	31.22	121.04	*S. canadensis*	*S. canadensis*	4x
CN16	31.49	120.42	*S. canadensis*	*S. canadensis*	6x
CN17	34.44	119.24	*S. canadensis*	*S. canadensis*	4x/6x
CN18	31.93	120.02	*S. canadensis*	*S. canadensis*	6x
CN20	32.09	119.24	*S. canadensis*	*S. canadensis*	6x
CN21	32.13	119.33	*S. canadensis*	*S. canadensis*	6x
CN22	33.97	118.31	*S. canadensis*	*S. canadensis*	6x
CN23	33.33	120.15	*S. canadensis*	*S. canadensis*	6x
CN24	34.31	117.21	*S. canadensis*	*S. canadensis*	6x
CN25	31.83	120.27	*S. canadensis*	*S. canadensis*	6x
CN26	33.18	120.35	*S. canadensis*	*S. canadensis*	4x
CN27	27.47	120.37	*S. canadensis*	*S. canadensis*	6x
CN28	29.06	119.39	*S. canadensis*	*S. canadensis*	6x
CN29	30.45	120.44	*S. canadensis*	*S. canadensis*	4x
CN30	29.05	121.37	*S. canadensis*	*S. canadensis*	4x
CN31	30.18	120.19	*S. canadensis*	*S. canadensis*	6x
CN32	30.16	120.13	*S. canadensis*	*S. canadensis*	4x/6x
CN33	28.62	119.30	*S. canadensis*	*S. canadensis*	6x
CN34	28.57	121.31	*S. canadensis*	*S. canadensis*	6x
CN35	28.95	118.87	*S. canadensis*	*S. canadensis*	6x
CN36	29.99	120.54	*S. canadensis*	*S. canadensis*	6x
CN38	30.88	120.17	*S. canadensis*	*S. canadensis*	6x
CN39	30.56	117.48	*S. canadensis*	*S. canadensis*	6x
CN40	29.52	121.37	*S. canadensis*	*S. canadensis*	6x
CN41	32.55	117.22	*S. canadensis*	*S. canadensis*	6x
CN42	31.47	117.18	*S. canadensis*	*S. canadensis*	6x
CN43	32.40	117.04	*S. canadensis*	*S. canadensis*	6x

CN44	31.22	118.22	*S. canadensis*	*S. canadensis*	4x
CN45	30.25	116.39	*S. canadensis*	*S. canadensis*	6x
CN46	30.70	118.91	*S. canadensis*	*S. canadensis*	6x
CN47	31.59	116.52	*S. canadensis*	*S. canadensis*	6x
CN48	29.74	116.04	*S. canadensis*	*S. canadensis*	6x
CN49	28.73	115.88	*S. canadensis*	*S. canadensis*	6x
CN50	29.35	117.19	*S. canadensis*	*S. canadensis*	4x
CN53	29.35	113.09	*S. canadensis*	*S. canadensis*	6x
CN54	27.85	113.10	*S. canadensis*	*S. canadensis*	6x
CN55	27.10	112.88	*S. canadensis*	*S. canadensis*	6x
CN56	28.26	113.05	*S. canadensis*	*S. canadensis*	6x
CN58	30.74	111.25	*S. canadensis*	*S. canadensis*	6x
CN59	31.62	111.17	*S. canadensis*	*S. canadensis*	6x
CN60	34.79	113.56	*S. canadensis*	*S. canadensis*	6x
CN61	27.73	118.01	*S. canadensis*	*S. canadensis*	6x
CN62	25.62	110.65	*S. canadensis*	*S. canadensis*	4x/6x
CN64	31.47	120.25	*S. canadensis*	*S. canadensis*	4x
CN65	32.11	118.88	*S. canadensis*	*S. canadensis*	4x
CN69	31.97	120.64	*S. canadensis*	*S. canadensis*	6x
RUS01	43.12	131.89	*S. canadensis*	*S. canadensis*	2x
EU01	6.76	51.43	*S. canadensis*	*S. canadensis*	2x
EU02	7.46	51.37	*S. canadensis*	*S. canadensis*	2x
EU03	7.46	51.37	*S. canadensis*	*S. canadensis*	2x
EU09	2.12	48.81	*S. canadensis*	*S. canadensis*	6x
EU10	13.37	52.51	*S. canadensis*	*S. canadensis*	6x
EU11	12.49	41.89	*S. canadensis*	*S. canadensis*	6x
EU12	11.27	43.78	*S. canadensis*	*S. canadensis*	6x
EU13	16.38	48.19	*S. canadensis*	*S. canadensis*	6x
EU14	7.53	47.02	*S. canadensis*	*S. canadensis*	2x
EU15	7.75	47.22	*S. canadensis*	*S. canadensis*	2x
JP01	36.23.59	140.40.29	*S. altissima*	*S. canadensis*	6x
JP02	36.27.59	140.39.29	*S. altissima*	*S. canadensis*	6x
JP03	36.37.08	140.20.07	*S. altissima*	*S. canadensis*	6x
JP04	36.34.92	140.20.52	*S. altissima*	*S. canadensis*	6x
JP05	36.33.49	140.51.80	*S. altissima*	*S. canadensis*	6x
Cultivated *S.canadensis* in China					
LYG	34.60	119.18	*S. canadensis* (	*S. canadensis*	2x
NJ	32.08	118.79	*S. canadensis*	*S. canadensis*	2x
YN	25.07	102.76	*S. canadensis*	*S. canadensis*	2x
Native range					
CA09	45.50	-75.80	*S. canadensis*	*S. canadensis*	2x
CA13	46.31	-72.65	*S. canadensis*	*S. canadensis*	2x
CA14	46.76	-71.29	*S. canadensis*	*S. canadensis*	2x
US02	34.25	-79.70	*S. canadensis*	*S. canadensis*	4x/6x

US03	34.23	-77.83	*S. canadensis*	*S. canadensis*	4x/6x
US04	37.80	-81.22	*S. canadensis*	*S. canadensis*	6x
US06	38.35	-81.65	*S. canadensis*	*S. canadensis*	4x/6x
US07	37.15	-79.23	*S. canadensis*	*S. canadensis*	4x/6x
US08	36.97	-81.07	*S. canadensis*	*S. canadensis*	4x
US09	36.18	-84.07	*S. canadensis*	*S. canadensis*	4x
US10	35.05	-84.90	*S. canadensis*	*S. canadensis*	6x
US12	40.24	-76.80	*S. canadensis*	*S. canadensis*	4x/6x
US13	41.08	-75.96	*S. canadensis*	*S. canadensis*	6x
US14	41.38	-75.03	*S. canadensis*	*S. canadensis*	4x/6x
US23	41.42	-73.34	*S. canadensis*	*S. canadensis*	6x
US25	41.49	-71.54	*S. canadensis*	*S. canadensis*	6x
US27	42.45	-71.24	*S. canadensis*	*S. canadensis*	4x/6x
US28	43.03	-73.79	*S. canadensis*	*S. canadensis*	4x/6x
US29	43.14	-77.67	*S. canadensis*	*S. canadensis*	4x/6x
US30	43.09	-79.00	*S. canadensis*	*S. canadensis*	4x/6x
US31	42.47	79.32	*S. canadensis*	*S. canadensis*	6x
US34	42.92	87.94	*S. canadensis*	*S. canadensis*	4x/6x
US35	39.10	84.28	*S. canadensis*	*S. canadensis*	6x
US36	39.89	84.17	*S. canadensis*	*S. canadensis*	6x
US40	39.47	82.31	*S. canadensis*	*S. canadensis*	4x/6x
US20	42.05	-80.08	*S. altissima*	*S. canadensis*	6x
US33	43.17	-85.63	*S. altissima*	*S. canadensis*	6x
US52	43.64	73.24	*S. canadensis*	*S. canadensis*	4x/6x
US53	43.98	72.63	*S. canadensis*	*S. canadensis*	2x
US54	43.94	72.63	*S. canadensis*	*S. canadensis*	4x/6x
US45	40.47	-86.93	*S. altissima*	*S. canadensis*	6x
US58	44.95	92.93	*S. canadensis*	*S. canadensis*	4x
US59	45.54	94.27	*S. canadensis*	*S. canadensis*	4x/6x
US60	45.61	95.88	*S. canadensis*	*S. canadensis*	4x
Closely related species					
US18	41.37	-75.06	*S. gigantea*	*S. gigantea*	
US51	43.35	-73.68	*S. gigantea*	*S. gigantea*	
US57	45.86	-96.73	*S. gigantea*	*S. gigantea*	
SS	45.50	-75.80	*S. simplex*	*S. simplex*	
SD	32.12	118.35	*S.decurrens*	*S.decurrens*	

Note：Blue represents Europe populations; green represents populations identified previously as *S. altissima*.

Table S2. List of distinct localities of *S. canadensis* and *S. altissima* with previously published ploidy level determination.

	Diploid	Tetraploid	Hexaploid
**Native rang (North America)**	359*	93	294
Abhilasha, D. et al. (2008)	4	2	0
Beaudry, J. R. & Chabot, D. L. (1959)	12	0	7
Beaudry, J. R. (1968)	10	0	0
Beaudry, J. R. & Chabot, D. L. (1957)	1	0	0
Beaudry, J. R. (1970)	3	0	0
Calder, J. A (1968)	0	1	0
Chinnappa, C. C. & Chmielewski, J. G. (1987)	1	0	0
Halverson, K. et al. (2008)	159	62	115
Keil, D. J. & Pinkava, D. J. et al. (1979)	1	0	0
Kapoor, B. M. (1970)	1	0	0
Kapoor, B. M. (1978)	2	0	0
Löve, Á. & Löve, D (1982)	2	0	0
Melville, M. R. & Mortorn, J. K. (1982)	98	0	82
Pringle, J. S. (1979)	1	0	0
Raven, P. H. et al. (1960)	0	0	1
Semple, J. C. et al. (1984)	2	7	12
Semple, J. C. et al. (1989)	16	2	2
Semple, J. C. & Chmielewski, J. G. (1987)	3	7	2
Semple, J. C. (1985)	3	2	6
Semple, J. C. et al. (1992)	1	3	2
Semple, J. C. et al. (1993)	2	2	0
Semple, J. C. et al. (2001)	4	5	9
Semple, J. C. & Cook (2004)	5	0	0
Tomkins D. J. & Grant, W. F. (1978)	8	0	0
van Kleunen, M. & Schmid, B. (2003)	17	0	51
Ward, D. E. & Spellenberg, R. W. (1986)	3	0	3
Ward, D. E. & Spellenberg, R. W. (1988)	0	0	2
**Introdued range**	150	0	2
Switzerland (Abhilasha, D. et al. 2008; van Kleunen, M. & Schmid, B.,2003)	26	0	0
Poland (Malecka J.1988，Malecka J 1978, Musial, K.1989a, Malecka, J.1989b, van Kleunen, M. & Schmid, B. 2003, Morto J. K. et al. 2018, Musiał K. et al. 2020)	78	0	0
Russia (Probatova et al. 2008)	1	0	0
Germany (Cheng et al. 2020a, van Kleunen, M. & Schmid, B. 2003)	19	0	0

Czech Republic (van Kleunen, M. & Schmid, B. 2003)	8	0	0
France (van Kleunen, M. & Schmid, B. 2003)	8	0	0
Austria (van Kleunen, M. & Schmid, B. 2003)	8	0	0
Belgium (Verloove F. et al. 2017)	0	0	1
Holand (Xu H Q 2016)	1	0	0
UK (Semple J. C 2004)	1	0	0
India (Sarkar, A. K. et al.1980)	0	0	1

Note: Part of the literature does not explicitly mention the number of diploid plants, so the umber was the count to one at the collection sites.

Table S3. Test of ITS and *psb*A-*trn*H intergenic spacer sequences of *Solidago altissima* blasted in NCBI database

Population codes	Accession number from Genebank	Species	Query cover	Homology comparison to *S. canadensis*	Variable sites
ITS sequences					
JP01-JP04	HQ142590.1	*S. canadensis*	100%	100%	
US20	HQ142590.1	*S. canadensis*	100%	100%	
US33	HQ142590.1	*S. canadensis*	100%	100%	
US45	HQ142590.1	*S. canadensis*	100%	99.84%	5(C→T)
*psb*A-*trn*H intergenic spacer sequences					
JP01-JP04	KX214929.1	*S. canadensis*	91%	100%	
US20	KX214929.1	*S. canadensis*	91%	100%	
US33	KX214929.1	*S. canadensis*	100%	100%	
US45	KX214929.1	*S. canadensis*	100%	100%	

Table S4. Flow cytometric ploidy analysis of Europe populations

Population	Ratio of peak positions (Mean± SD)	Number of measurements	Ploidy levels
EU01	1	6	2
EU02	1	6	2
EU03	1	6	2
EU09	2.604±0.055	6	6
EU10	2.623±0.002	6	6
EU11	2.628±0.054	6	6
EU12	2.712±0.065	6	6
EU13	2.671±0.050	6	6
EU14	1	6	2
EU15	1	6	2

Note: the native diploid population (CA09) used as the internal reference.

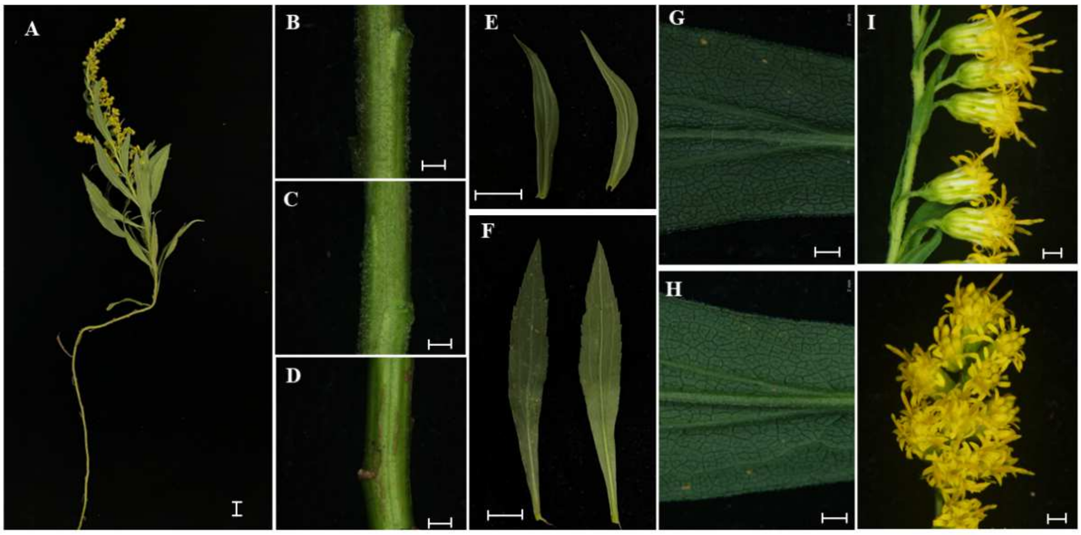

Figure S1. Morphology of *S. canadensis* (2x) form Canada. A. Shoot, scale = 1 cm; B-D, upper, middle, lower stem, scale = 2 mm; E-F, upper, lower stem leaves, scale = 1 cm; G-H, upper epidermal veins, lower epidermal veins, scale = 2 mm; I, involucres, scale = 2 mm; J, florets, scale = 2 mm.

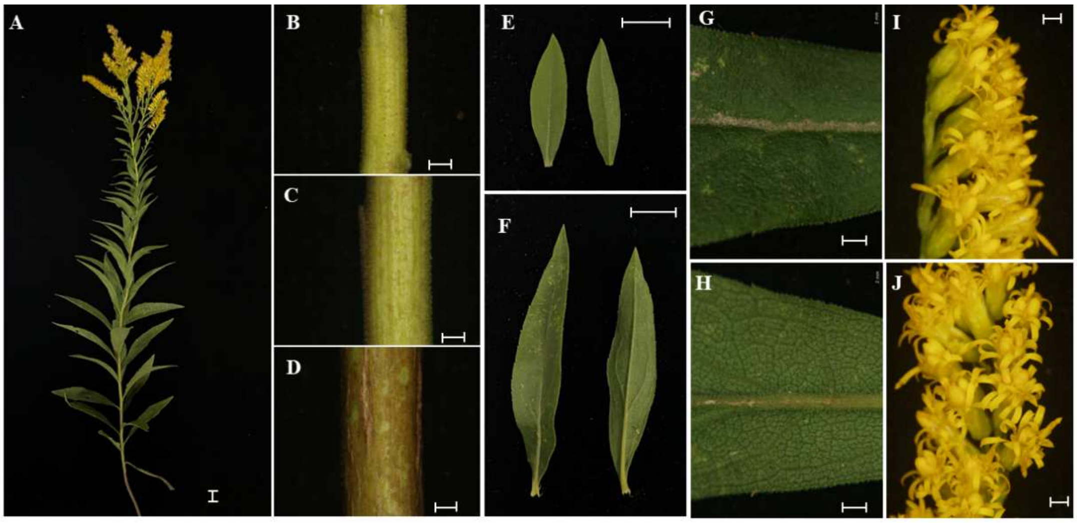

Figure S2. Morphology of *S. canadensis* (6x) form USA. A. Shoot, scale = 1 cm; B-D, upper, middle, lower stem, scale = 2 mm; E-F, upper, lower stem leaves, scale = 1 cm; G-H, upper epidermal veins, lower epidermal veins, scale = 2 mm; I, involucres, scale = 2 mm; J, florets, scale = 2 mm.

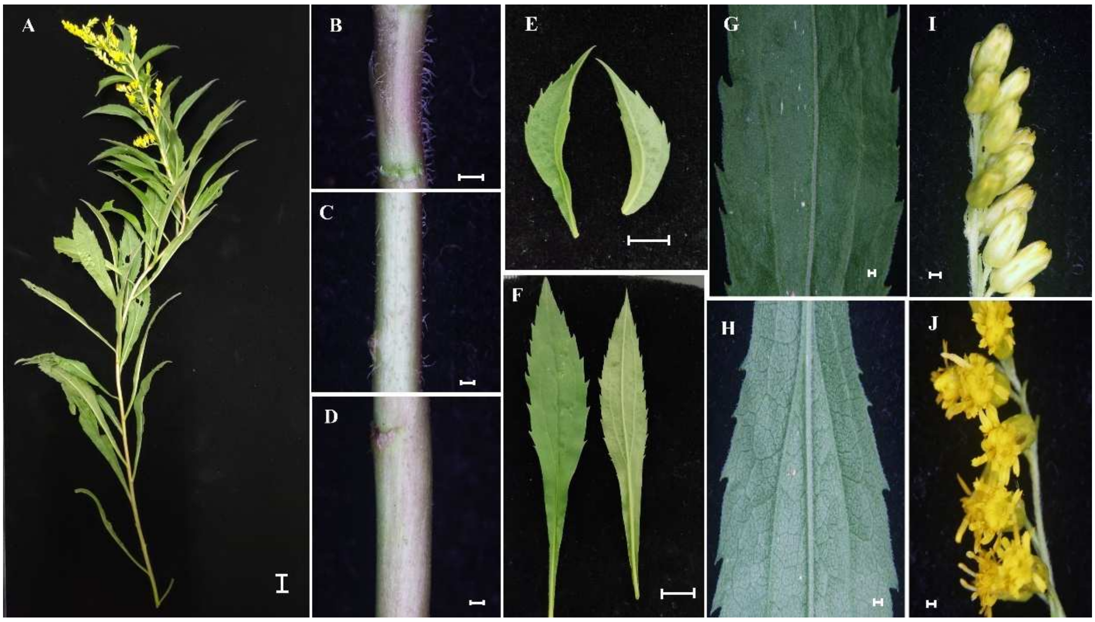

Figure S3. Morphology of *S. canadensis* (2x) form Russia. A. Shoot, scale = 1 cm; B-D, upper, middle, lower stem, scale = 2 mm; E-F, upper, lower stem leaves, scale = 1 cm; G-H, upper epidermal veins, lower epidermal veins, scale = 1 mm; I, involucres, scale = 1 mm; J, florets, scale = 1 mm.

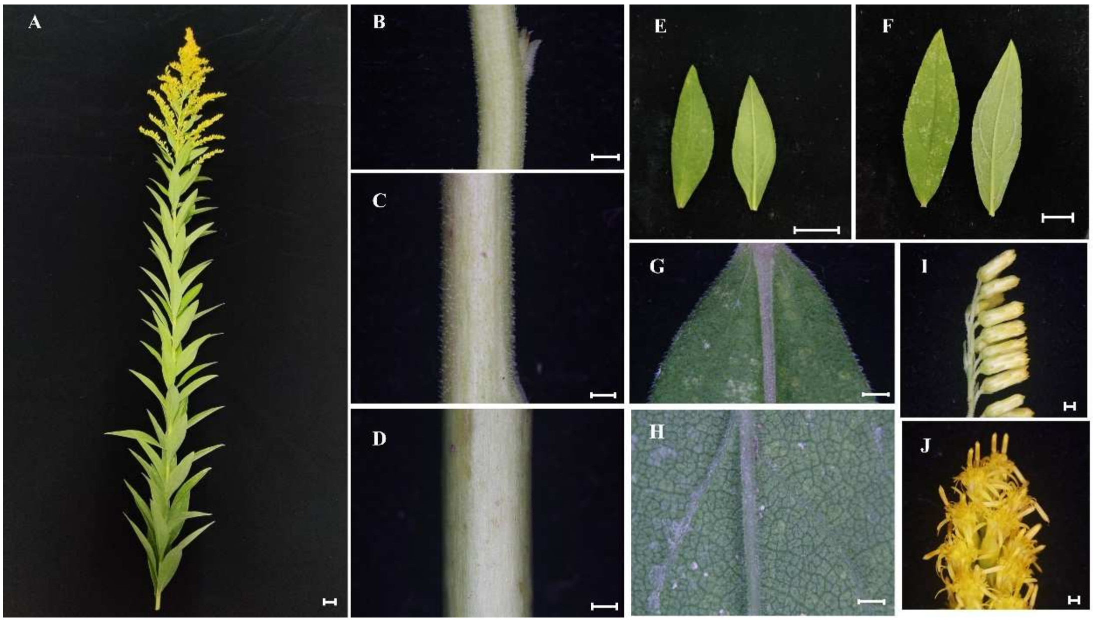

Figure S4. Morphology of *S. canadensis* (6x) form China. A. Shoot, scale = 1 cm; B-D, upper, middle, lower stem, scale = 2 mm; E-F, upper, lower stem leaves, scale = 1 cm; G-H, upper epidermal veins, lower epidermal veins, scale = 1 mm; I, involucres, scale = 1 mm; J, florets, scale = 1 mm.

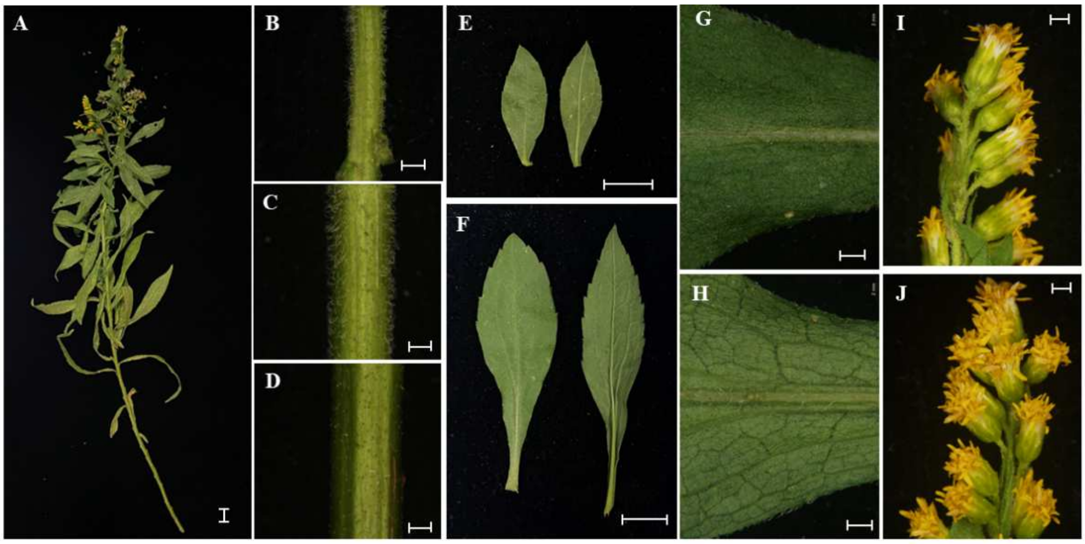

Figure S5. Morphology of *S. canadensis* (2x) form Europe. A. Shoot, scale = 1 cm; B-D, upper, middle, lower stem, scale = 2 mm; E-F, upper, lower stem leaves, scale = 1 cm; G-H, upper epidermal veins, lower epidermal veins, scale = 2 mm; I, involucres, scale = 2 mm; J, florets, scale = 2 mm.

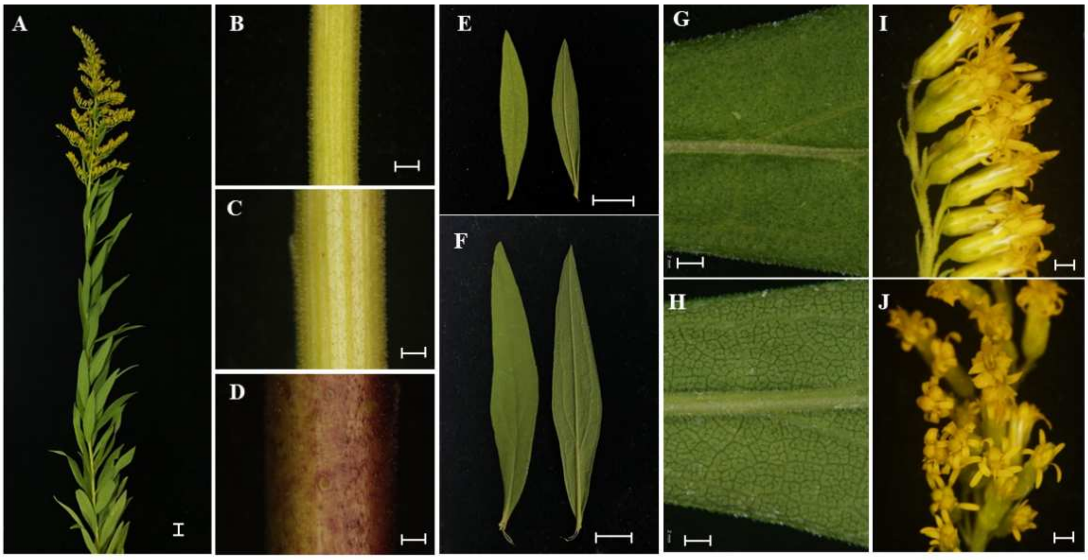

Figure S6. Morphology of *S. canadensis* (6x) form Europe. A. Shoot, scale = 1 cm; B-D, upper, middle, lower stem, scale = 2 mm; E-F, upper, lower stem leaves, scale = 1 cm; G-H, upper epidermal veins, lower epidermal veins, scale = 2 mm; I, involucres, scale = 2 mm; J, florets, scale = 2 mm.

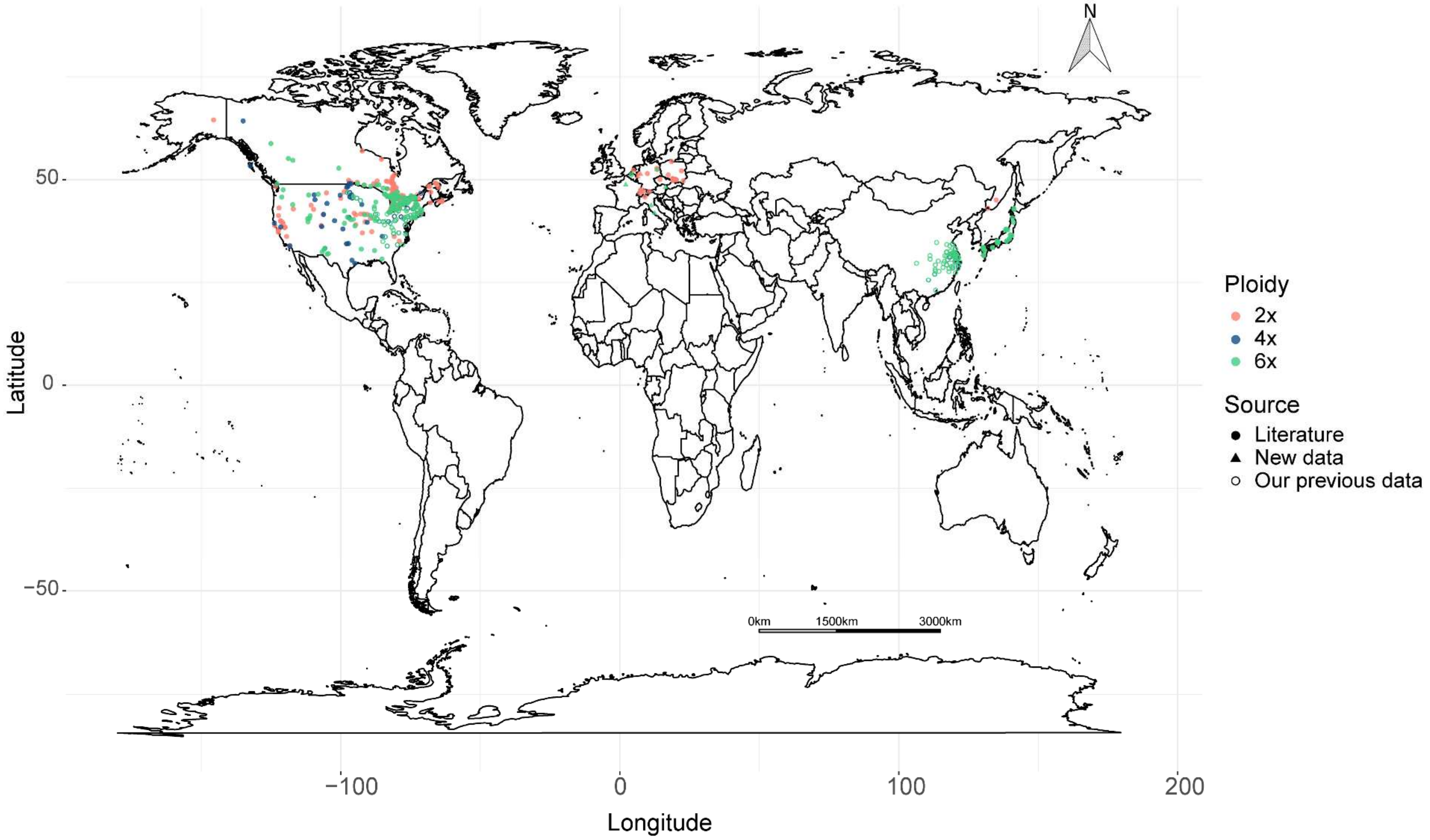

Figure S7. Cytogeography of *S. canadensis* at the Northern Hemisphere. The source of population information was shown in Table S2, our previous data was described in Cheng et al. [6].
